# Supplementary material for: Design principles of collateral sensitivity-based dosing strategies
Source: Nat Commun. 2021 Sep 28;12:5691. doi: 10.1038/s41467-021-25927-3 (PMC8479078; doi:10.1038/s41467-021-25927-3)
Supplement: Supplementary file 2 — Description of Additional Supplementary Files [file 41467_2021_25927_MOESM2_ESM.pdf]

Title: Supplementary Software 1

Description: This .zip file contains all files needed to recreate the results in the study, as well as a minimal quick demonstration scenario. The model and simulation framework is built in the statistical software R. The model framework script (CS_model_function.R) is needed to run the model which can be done using the demo script (Scenario_1_DEMO.R) or any of the execution scrips (Scenario_XX.R). The plot script (Plot_script.R) is need to generate the figures using the respective figure scripts. A detailed overview and description can be found the included readme file.
