# Supplementary material for: Design principles of collateral sensitivity-based dosing strategies
Source: Nat Commun. 2021 Sep 28;12:5691. doi: 10.1038/s41467-021-25927-3 (PMC8479078; doi:10.1038/s41467-021-25927-3)
Supplement: Supplementary file 3 — Supplementary Software [file 41467_2021_25927_MOESM3_ESM.zip › Aulin_NCOMM_2021_Results/Readme.pdf]

1. System requirements

Software dependencies and operating systems (including version numbers)

Operating system:	Windows 10
R version:	4.0.5

Required R packages and versions tested:

Model function:

RxODE	v 1.0.0.0
dplyr	v 1.0.5
tidyr	v 1.1.3
doParallel	v 1.0.16
doRNG	v 1.8.2

Post processing and data visualization:

dplyr	v 1.0.5
ggplot2	v 3.3.3
forcats	v 0.5.1
patchwork	v 1.1.1

2. Installation guide

No additional installation needed other than R, R studio and required packages. For installation of R packages available at CRAN use command install.packages(”package_name”). For further information refer to CRAN documentation of specific packages. These dependence are all typically quick to install (<5 min).

3. File overview (38 files, 5 folders)

- *CS_model_function.R* : The script contains the CS model function (CS_model()), which simulates bacterial dynamics relating to treatment of an infection in *n* number of patients treated with two antibiotics administrated i.v. twice daily according to different combination treatment schedules. The function can be run without any input arguments, and will this result in the default parameter settings. To run a set of simulations with different input parameters we recommend to use the an execution script.

- *Plot_script.R*: Script containing data visualization functions to be used for generating figures.

- **Demo**
  - *Scenario_1_DEMO.R*: Execution script used to run a demonstration of the CS model with 10 simulated patients.
  - *FIG_DEMO*.R*:* Script processing the simulation results generated with script *Scenario_1_DEMO.R* to make a bar plot of probability of resistance at end of treatment.
  - **Output_Demo**
    - *FIG_demo.pdf*: Bar plot generated using *FIG_DEMO*.R
    - **Scenario_1**
      - *Scenario_1_input.rsd*: Input parameters for Scenario_1
      - *Scenario_1_sim_xxx.rsd:* 12 files of simulated data generated using *Scenario_1_DEMO.R*
- **Execution_Scripts**
  - *Scenario_xx.R:* 10 execution script used to run the scenarios outlined in Table 1.
- **Figure_Scripts**
  - *FIGx_xx.R:* 9 script used generated the figures included in the manuscript, includes both main figure and corresponding supplemental figure when applicable.

4. Demonstration

We have included a small demonstration on how to use the model framework. This demonstration simulates 12 simulations with different input parameters, each simulated for 10 patients using. The simulation includes simulations using different types of drugs and with and without reciprocal collateral sensitivity. To run the demonstration use the script named *Scenario_1_DEMO.R* found in the in the *Demo* folder. Note that this script will source the CS model script *CS_model_function.R*. This script (if not altered) will generate a folder, named *Scenario_1*, containing 13 .RDS files, one of which includes the input parameters (*Scenario_1_input.rsd*) and the other 12 contain simulated data of the different simulations. The runtime of this demonstration approximately 25 minutes on an i7 laptop.

To process the simulated data use the *FIG_DEMO.R* script in the *Demo* folder. This will generate a PDF with a bar plot similar to those included in the manuscript. Note that this script will source the figure script *Plot_script.R*.

For reference all generated demo files are included in the *Output_Demo* folder.

5. Instructions for use and replication of results for manuscript

The scripts for all simulations included in the manuscript can be found in the *Execution _Scripts* folder. A summary of all scenarios and their corresponding figures can be found in **Table 1**. All figure scripts are named with their figure number and can be found in the *Figure_Scripts* folder and are often based on several Scenarios.

To generate a figure, first run all execution scripts related to the figure to simulate the data, then run the figure script of interest.

Table 1. Overview of simulation scenarios and figures

Scenario	N different simulations	Description	Figure
38	32	dose finding with mono A	S1
39	12	base scenario	4,5,6,8,9,10, S1, S2, S3, S5
40	24	reciprocity	5, S2
41	12	different drugs	6, S3
42	96	Steady state concentration (Css)	7, S4
43	60	fitness	8
46	60	fitness	8
47	32	Pre-existing resistance	9, S5
48	60	mutation rate	10
49	12	different drugs	6
