# Supplementary figures and images for: Design principles of collateral sensitivity-based dosing strategies
Source: Nat Commun. 2021 Sep 28;12:5691. doi: 10.1038/s41467-021-25927-3 (PMC8479078; doi:10.1038/s41467-021-25927-3)

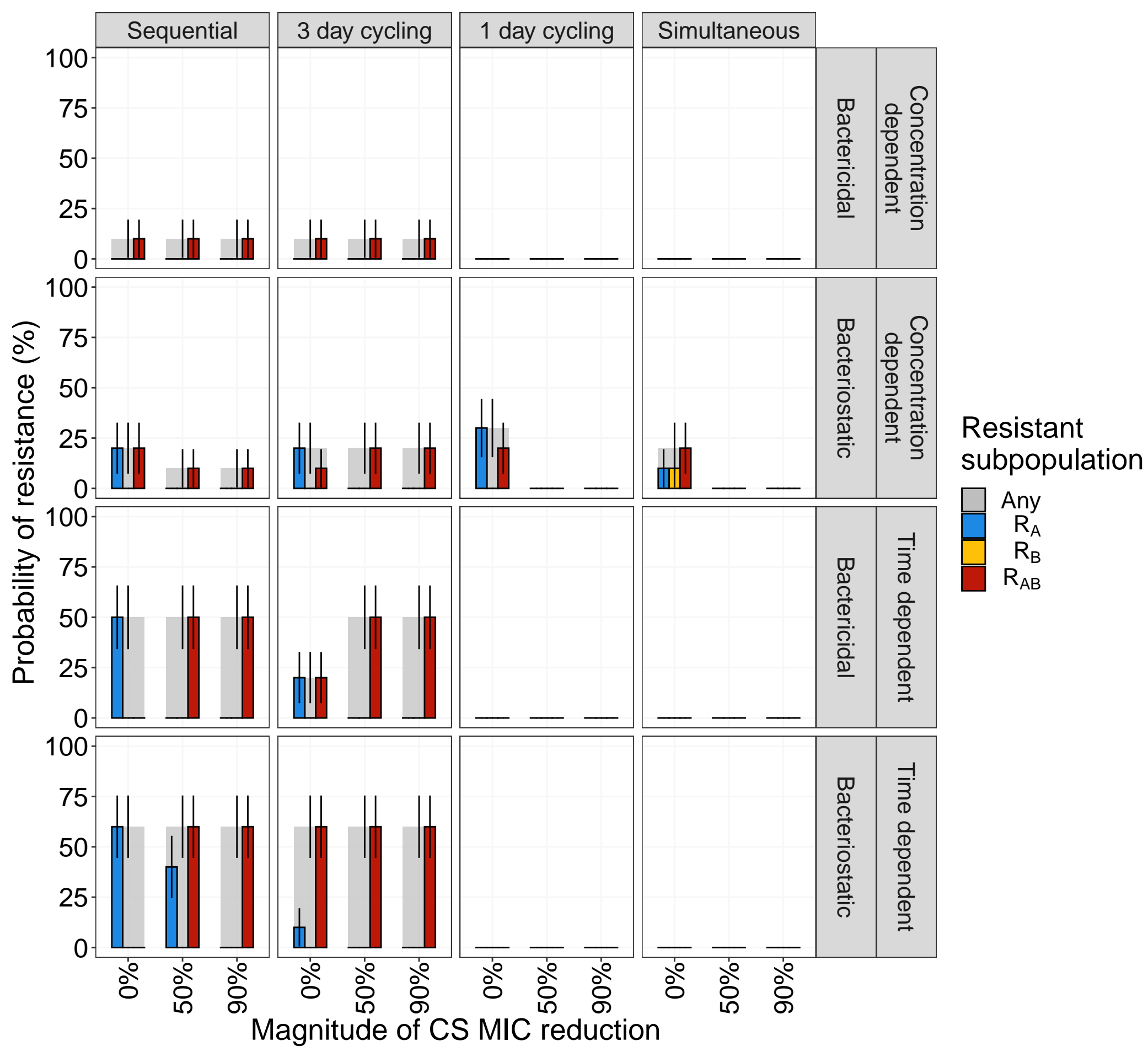

Supplement: Supplementary file 3 — Supplementary Software [file 41467_2021_25927_MOESM3_ESM.zip › Aulin_NCOMM_2021_Results/Demo/Output_Demo/FIG_demo.pdf]
